# Knowledge mapping of exosomes in metabolic diseases: a bibliometric analysis (2007-2022)

**DOI:** 10.3389/fendo.2023.1176430

**Published:** 2023-05-08

**Authors:** Fangzhi Xu, Chenxi Xia, Lin Dou, Xiuqing Huang

**Affiliations:** ^1^ The Key Laboratory of Geriatrics, Beijing Institute of Geriatrics, Institute of Geriatric Medicine, Chinese Academy of Medical Sciences, Beijing Hospital, National Center of Gerontology of National Health Commission, Beijing, China; ^2^ Department of Cardiology, Beijing Hospital, National Center of Gerontology, Institute of Geriatric Medicine, Chinese Academy of Medical Sciences, Beijing, China

**Keywords:** exosomes, metabolic diseases, bibliometric, knowledge-map, citespace, VOSviewer, bibliometrix

## Abstract

**Background:**

Research on exosomes in metabolic diseases has been gaining attention, but a comprehensive and objective report on the current state of research is lacking. This study aimed to conduct a bibliometric analysis of publications on “exosomes in metabolic diseases” to analyze the current status and trends of research using visualization methods.

**Methods:**

The web of science core collection was searched for publications on exosomes in metabolic diseases from 2007 to 2022. Three software packages, VOSviewer, CiteSpace, and R package “bibliometrix” were used for the bibliometric analysis.

**Results:**

A total of 532 papers were analyzed, authored by 29,705 researchers from 46 countries/regions and 923 institutions, published in 310 academic journals. The number of publications related to exosomes in metabolic diseases is gradually increasing. China and the United States were the most productive countries, while Ciber Centro de Investigacion Biomedica en Red was the most active institution. *The International Journal of Molecular Sciences* published the most relevant studies, and *Plos One* received the most citations. Khalyfa, Abdelnaby published the most papers and Thery, C was the most cited. The ten most co-cited references were considered as the knowledge base. After analysis, the most common keywords were microRNAs, biomarkers, insulin resistance, expression, and obesity. Applying basic research related on exosomes in metabolic diseases to clinical diagnosis and treatment is a research hotspot and trend.

**Conclusion:**

This study provides a comprehensive summary of research trends and developments in exosomes in metabolic diseases through bibliometrics. The information points out the research frontiers and hot directions in recent years and will provide a reference for researchers in this field.

## 1 Introduction

Metabolic diseases are a group of diseases caused by abnormalities in amino acid and glucolipid metabolism within the body. These conditions are influenced by various factors such as genetics, environment, and lifestyle. Metabolic diseases encompass a wide range of illnesses, including but not limited to, obesity, type 2 diabetes(T2D), insulin resistance(IR), hyperlipidemia, non-alcoholic fatty liver disease(NAFLD), atherosclerosis(AS), and metabolism-related cancers (1). In recent years, unhealthy dietary patterns and sedentary lifestyles, attributable to improving living standards, have contributed to the rising of metabolic diseases (2). Therefore, metabolic diseases have become an critical factor threatening human health globally.

Exosomes are small vesicles, 30-200 nm in diameter, that are released into the extracellular space by multivesicular bodies. They contain various biologically active substances such as proteins, nucleic acids, lipids, and enzymes. These substances exert their effects on neighboring target cells through autocrine or paracrine mechanisms or on specific, distant target cells through humoral transport. Subsequently, exosomes interact directly with target cells by membrane fusion or endocytosis, exhibiting complex functions in intercellular communication and compound exchange, which play important roles in human health and disease (3). Recent research has found that the number, contents and metabolism of exosomes are strongly associated with the occurrence and progression of metabolic diseases. Therefore, the use of exosomes in the treatment of metabolic diseases is a promising avenue of research (4).

Bibliometrics is a literature analysis method that evaluates the intrinsic connections and distribution patterns among research literature from quantitative and qualitative perspectives. Its objective is to gain insights into the current status of research, research hotspots, and future trends in a specific field (5). When combined with visualization analysis, bibliometrics becomes an effective tool for integrating information and enhancing understanding of the research process (6). Therefore, this article presents a bibliometric analysis of the relevant literature on exosomes in metabolic diseases obtained from the Web of Science Core Collection(WoSCC) database from January 2007 to December 2022. This analysis intends to provide directions for future research.

## 2 Materials and methods

### 2.1 Data collection

**Figure 1:**
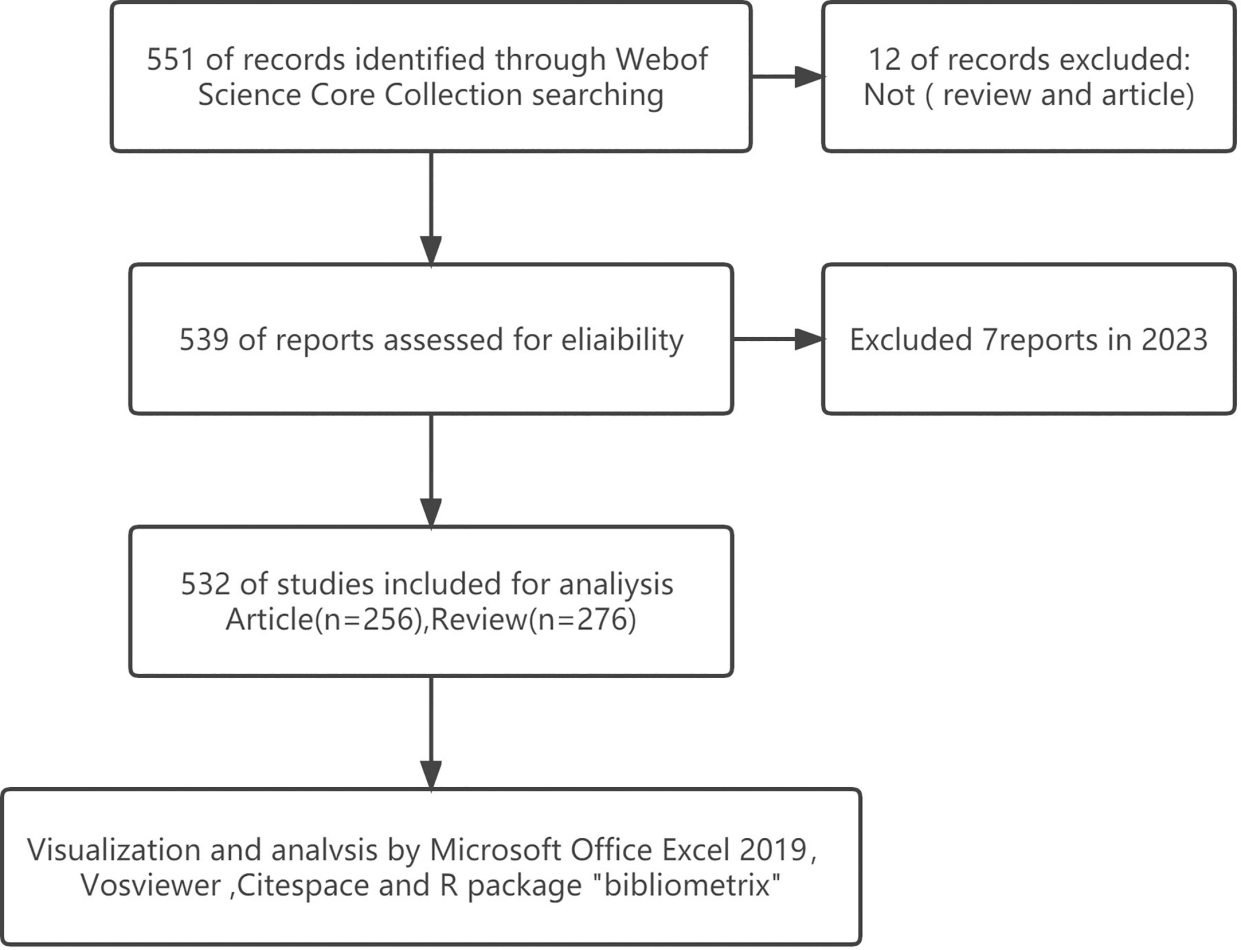
Flow chart of the data collection for research on exosomes in metabolic diseases.

We conducted a literature search on the WoSCC database on *6 February 2022*. And the retrieval strategy was [TS = (“exosome” OR “exosomes” AND “metabolic diseases”)] AND [article type = (article AND reviews)] AND [Time span = (January 2007 to December 2022)]. Publicly available data sets were analyzed in this study, and ethics statement was not required. A total of 532 items matching the search criteria were located and further analyzed (**Figure 1**).

### 2.2 Data analysis

VOSviewer9(version 1.6.18), CiteSpace(version 6.1.R3), Excel(version 2019), and R package “bibliometrix”(version 3.2.1) were used to perform bibliometric analysis and visualization.

VOSviewer is a bibliometric analysis software adept at creating and visualizing knowledge maps to extract critical information from numerous publications, often used to construct collaborative, co-citation, and co-occurrence networks (7). In our study, the software accomplished the following analyses: country and institution analysis, author and co-citation author analysis journal and co-citation journal analysis, and keyword co-occurrence analysis.

CiteSpace is a bibliometric and visual analysis tool for detecting collaborations, internal structures, key points, potential trends, and dynamics in a scientific field (8). In this study, we mainly use CiteSpace to analyze bursts, clustering, and timelines of keywords.

In addition, we analyzed trend topics and word growth using the R package “bibliometrix” (9), and publications were analyzed quantitatively using Microsoft Office Excel. Moreover, the Impact Factor (IF) and Journal Citation Reports (JCR) departments of the journals were obtained from Web of Science on February 6, 2023.

## 3 Results

### 3.1 Publication trend

**Figure 2:**
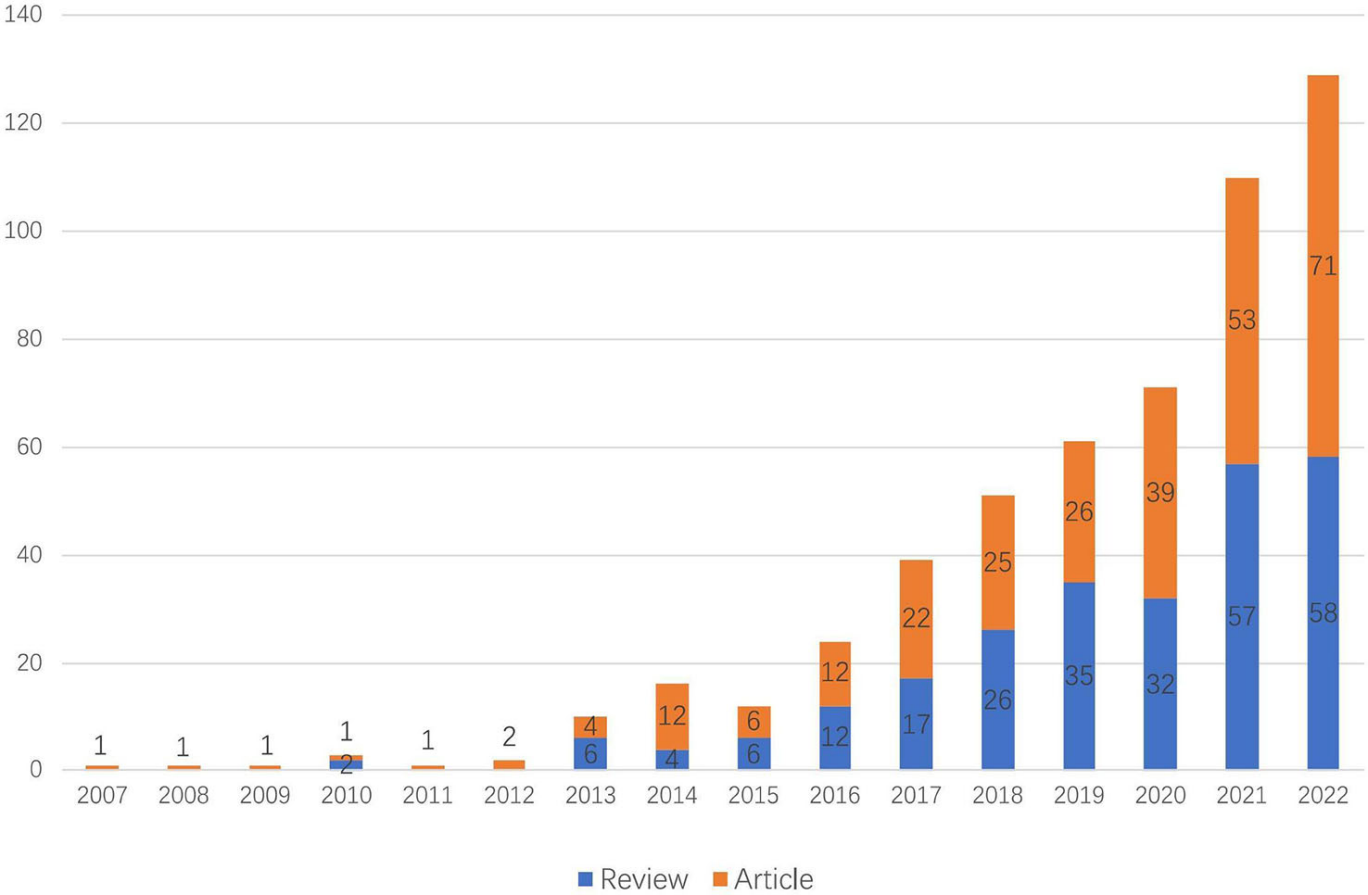
Trends of exosomes in metabolic diseases publications from 2007 to 2022.

As of 2022, there are 532 articles related to exosomes in metabolic diseases, including 255 reviews and 277 articles. As shown in **Figure 2**, the first relevant literature was published in 2007, and in the following six years (2007 - 2012), there were few publications, with an average of 1.5 publications per year, which was at the initial stage of exosome research in metabolic diseases. The period of 2013 - 2022 saw a significant increase in the number of publications, with an average of 53.2 publications per year; especially in 2015 - 2021, the number of publications showed an exponential rise, indicating that exosomes are gradually becoming a hot spot in the field of metabolic diseases.

### 3.2 Country and institutional distribution analysis

These publications were from 46 countries/regions and 923 institutions. As shown in **Table 1**, among the top 10 regions/countries and institutions engaged in exosome research in metabolic diseases, China ranked first with 166 publications, followed by the United States (159 publications), Italy (49 publications), Spain (34 publications), and Australia (31 publications); the top five institutions with the most publications were located in Spain, the United States, China, and France, with Ciber Centro de Investigacion Biomedica en Red (18 publications) was the most prolific institution, followed by the University of California System (15 publications), Chinese Academy of Sciences, Harvard University, and Institut National de la Sante et de la Recherche Medicale Inserm, all with 13 publications.

**Table 1:** Top 10 countries/regions and institutions related to exosomes in metabolic diseases research.

Country/ Region	Count	Percent (%)	Institute	Count	Percent (%)
China	166	31.203	Ciber Centro de Investigacion Biomedica en Red	18	3.383
USA	159	29.887	University of California System	15	2.82
Italy	49	9.211	Chinese Academy of Sciences	13	2.444
Spain	34	6.391	Harvard University	13	2.444
Australia	31	5.827	Institut National de la sante et de la Recherche Medicale Inserm	13	2.444
England	25	4.699	National Institutes of Health NIH USA	12	2.256
Germany	23	4.323	Shandong University	11	2.068
Japan	21	3.947	Shanghai Jiao Tong University	11	2.068
France	20	3.759	University of Queensland	11	2.068
Canada	17	3.195	Us Department of Veterans Affairs	11	2.068

**Figure 3:**
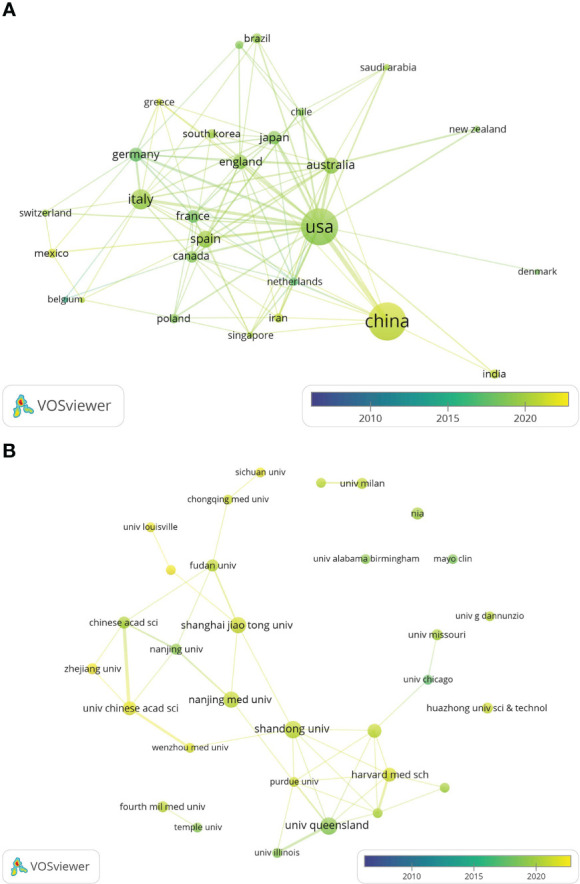
The distribution of countries and institutions publishing research on exosomes in metabolic diseases. **(A)** The network map of countries/regions publishing research on exosomes in metabolic diseases by VOSviewer. **(B)** The network map of institutes involved in research on exosomes in metabolic diseases by VOSviewer.

The global country and institutional distribution network of publications was visualized by using VOSviewer, and the association strength method was used for normalization. According to this national collaboration network (**Figure 3A**), we can find a lot of active cooperation among different countries. For example, China has close collaboration with the United States, Singapore and the Netherlands, and the United States has active collaboration with Australia, Italy and Japan. And the network map of the institution’s publications (**Figure 3B**) shows that the University of Queensland established collaborations with the University of Illinois and Massachusetts general hospital on the topic of exosomes in metabolic diseases in the early period, while 11 universities from China(Chinese Academy of Sciences, Shandong University, Shanghai Jiao Tong University, etc.) have collaborated closely on this topic in the last five years.

### 3.3 Journal distribution analysis

**Figure 4:**
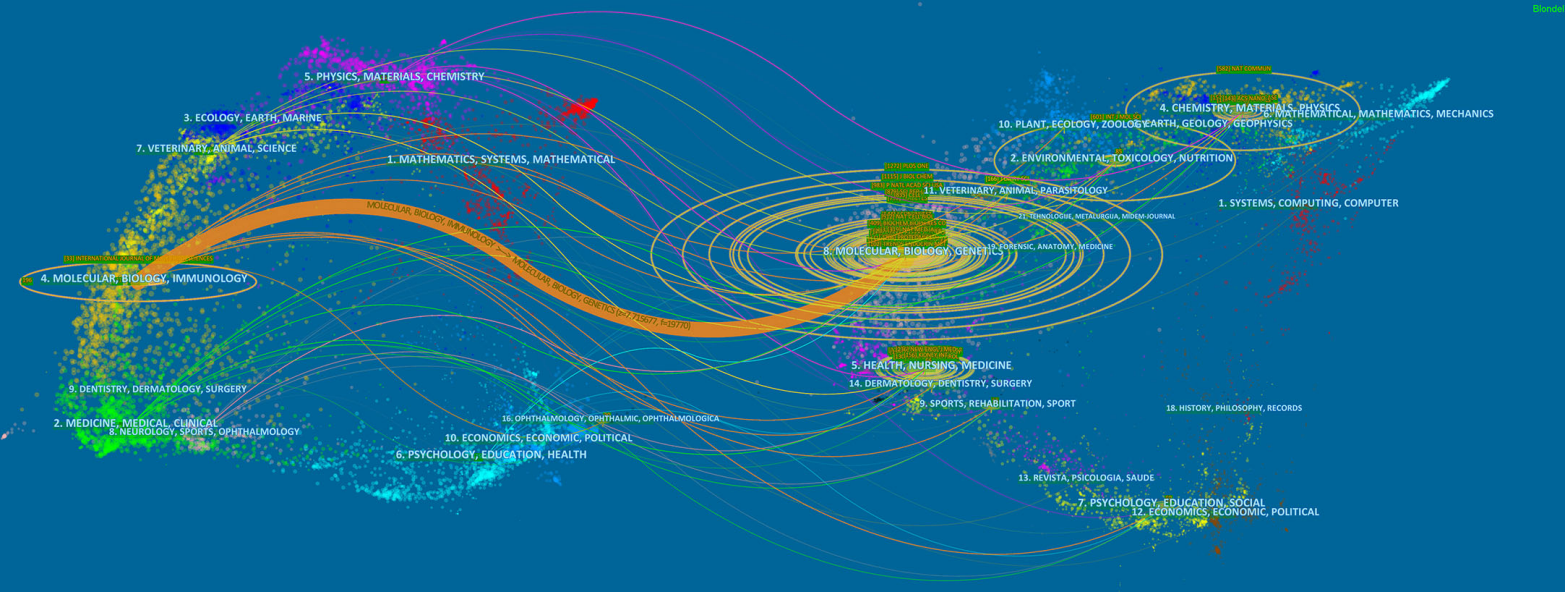
A dual-map overlay of journals related to the exosomes in metabolic diseases from 2007 to 2022.

Papers on exosomes in metabolic diseases were published in 310 journals. As shown in **Table 2**, *International Journal of Molecular Sciences* published the most studies with 33 articles, followed by *Frontiers in Endocrinology* and *Frontiers in Immunology*, both with 16 articles. In 2022, the impact factors of these journals ranged from 4.7 to 11.6, with *Theranostics* having the highest impact factor and *Plos One* having the lowest impact factor. According to the JCR partition analysis, Q1 accounts for 50% of this ranking, and Q2 accounts for 50%. Besides, the top 10 co-cited journals were all cited more than 500 times, with *Plos One* (1272 citations) and *Journal of Biological Chemistry* (1115 citations) being the most cited, followed by *Proceedings of The National Academy of Sciences of the United States of America* (cited 983 times) and *Nature* (cited 879 times). Among them, *Nature* had the highest impact factor (IF= 69.504, 2022), followed by *Nature Communications* (17.694, 2022). We use VOSviewer to visualize the journal network (**Supplementary Figure 1A**) and the co-citation network diagram (**Supplementary Figure 1B**), which shows active journal citation relationship and co-citation relationship. We also used CiteSpace to map the links between cited and cited journals (**Figure 4**) and found that the main citation paths were molecular, biology, immunology-molecular to molecular, biology, genetics (z=7.71, f=19770).

**Table 2:** Top 10 journals and co-cited journals for research of exosomes in metabolic diseases.

Journals	Documents	2022 IF	2022 Q	co-cited journals	co-citation	2022 IF	2022 Q
**International Journal of Molecular Sciences**	33	6.208	Q1	Plos One	1272	3.752	Q2
**Frontiers in Endocrinology**	16	6.055	Q1	Jounal of biological Chemistry	1115	5.486	Q2
**Frontiers in Immunology**	16	8.786	Q1	Proceedings of the National Academy of Sciences of The United States of America	983	9.661	Q1
**Cells**	11	7.666	Q2	Nature	879	69.504	Q1
**Frontiers in Physiology**	11	4.755	Q1	Scientific Reports	864	4.996	Q2
**Cancers**	9	6.575	Q1	Cell	836	66.85	Q1
**Scientific Reports**	8	4.996	Q2	Diabetes	792	9.337	Q1
**Plos One**	7	3.752	Q2	Journal of Extracellular Vesicles	791	17.331	Q1
**Frontiers in Oncology**	6	4.755	Q1	International Journal of Molecular Sciences	601	6.208	Q1
**Theranostics**	6	11.6	Q1	Nature Communications	582	17.694	Q1

### 3.4 Author distribution analysis

A total of 29705 authors were involved in exosome research in metabolic diseases. **Table 3** lists the most published and cited authors, Khalyfa, Abdelnaby from the University of Missouri Columbia, USA (7 papers), followed by Gozal, David, also from the University of Missouri Columbia, USA (5 papers), and Liu, Jing from Dalian Medical University, China (5 papers). The most cited author was Thery, Clotilde from Paris Sciences & Lettres-PSL University, France (169 citations), followed by Raposo, G from Institut Curie, France (114 citations). We also screened and mapped the author network (**Supplementary Figure 2A**) and the co-cited network (**Supplementary Figure 2B**) through VOSviewer, and found that there was active cooperation between authors and co-cited authors.

**Table 3:** The top authors in the field of exosomes in metabolic diseases ranked by publication and citation numbers.

Author(publications ≥ 5)	Count	Co-cited author(Citations ≥ 80)	Citation
Khalyfa, Abdelnaby	7	Thery, C	169
Gozal, David	5	Raposo, G	114
Liu, Jing	5	Valadi, H	104
Salomon, Carlos	5	Zhang, Y	104
Andriantsitohaina, Ramaroson	4	Ying, W	95
Melnik, Bodo C.	4	Van Niel, G	93
Qang, Qun	4	Colombo, M	91
Bernabei, Roberto	3	Kranendonk, Meg	83
Bruschi, Maurizio	3	Guay, C	80

### 3.5 Reference distribution analysis

**Table 4** lists the top 10 most frequently cited studies, all of which were cited more than 50 times. “Exosome-mediated transfer of mRNAs and microRNAs is a novel mechanism of genetic exchange between cells” (10), published by Hadi Valadi et al. in *Nature Cell Biology* in 2007, had the highest number of citations at 103.

**Table 4:** Top 10 documents in citation analysis of publications on exosomes in metabolic diseases.

Rank	Title	First author	Corresponding author	Source	Publication year	Total citation
1	Exosome-mediated transfer of mRNAs and microRNAs is a novel mechanism of genetic exchange between cells	Hadi Valadi	Jan O Lötvall	Nature Cell Biology	2007	103
2	Biogenesis, secretion, and intercellular interactions of exosomes and other extracellular vesicles	Marina Colombo	Clotilde Théry	Annual Review of Cell and Developmental Biology	2014	75
3	Adipose-derived circulating miRNAs regulate gene expression in other tissues	Thomas Thomou	C Ronald Kahn	Nature	2013	73
4	Extracellular vesicles: exosomes, microvesicles, and friends	Graça Raposo	Willem Stoorvogel	the Journal of Cell Biology	2013	72
5	Adipose Tissue Macrophage-Derived Exosomal miRNAs Can Modulate In Vivo and In Vitro Insulin Sensitivity	Wei Ying	Jerrold M Olefsky	Cell	2017	72
6	Adipose tissue exosome-like vesicles mediate activation of macrophage-induced insulin resistance	Zhong-bin Deng	Huang-Ge Zhang	Diabetes	2009	66
7	Shedding light on the cell biology of extracellular vesicles	Guillaume van Niel	Graça Raposo	Nature Reviews. Molecular Cell Biology	2017	58
8	Minimal information for studies of extracellular vesicles 2018 (MISEV2018): a position statement of the International Society for Extracellular Vesicles and update of the MISEV2014 guidelines	Clotilde Théry	Ewa K Zuba-Surma	Journal of Extracellular vesicles	2018	57
9	Biological properties of extracellular vesicles and their physiological functions	María Yáñez-Mó	Olivier De Wever	Journal of Extracellular vesicles	2015	52
10	Proteomic comparison defines novel markers to characterize heterogeneous populations of extracellular vesicle subtypes	Joanna Kowal	Clotilde Théry	Proceedings of the National Academy of Science of the United States of America	2016	51

**Figure 5:**
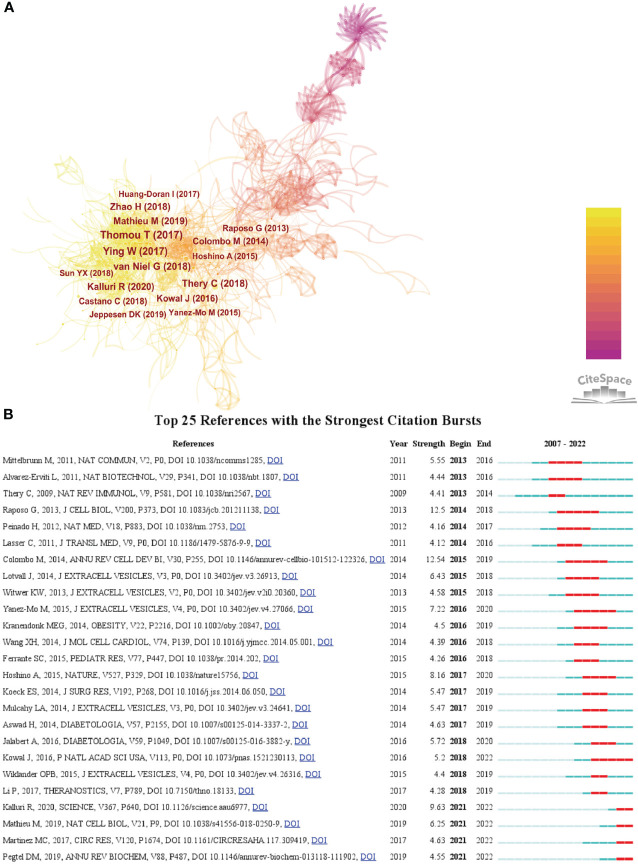
**(A)** The visualization of co-cited references on research of exosomes in metabolic diseases. **(B)** Top 25 references with strong citation bursts. A red bar indicates high citations in that year.

We then used CiteSpace to analyze and visualize the co-citation network of the top 16 publications shortlisted (**Figure 5A**), and it can be seen that “Adipose-derived circulating miRNAs regulating gene expression in other tissues” (11) published in *Nature* by Thomas Thomou in 2013 is the pivotal node in the co-citation network. **Figure 5B** shows the top 25 citations with strong citation bursts, and citation bursts for references appeared as early as 2013 and as late as 2021. The literature with the strongest citation burst is “Biogenesis, secretion, and intercellular interactions of exosomes and other extracellular vesicles” (12) (strength = 12.54) by Marina Colombo et al. in 2014 in *Annual Review of Cell and Developmental Biology*, with citation bursts from 2015 to 2019. Next is the article “Extracellular vesicles: exosomes, microvesicles, and friends” (13)(strength = 12.5) by Graça Raposo and Willem Stoorvogel in *Journal of Cell biology* in 2013, with a citation burst from 2014 to 2018.

### 3.6 Keyword co-occurrence cluster analysis

**Figure 6:**
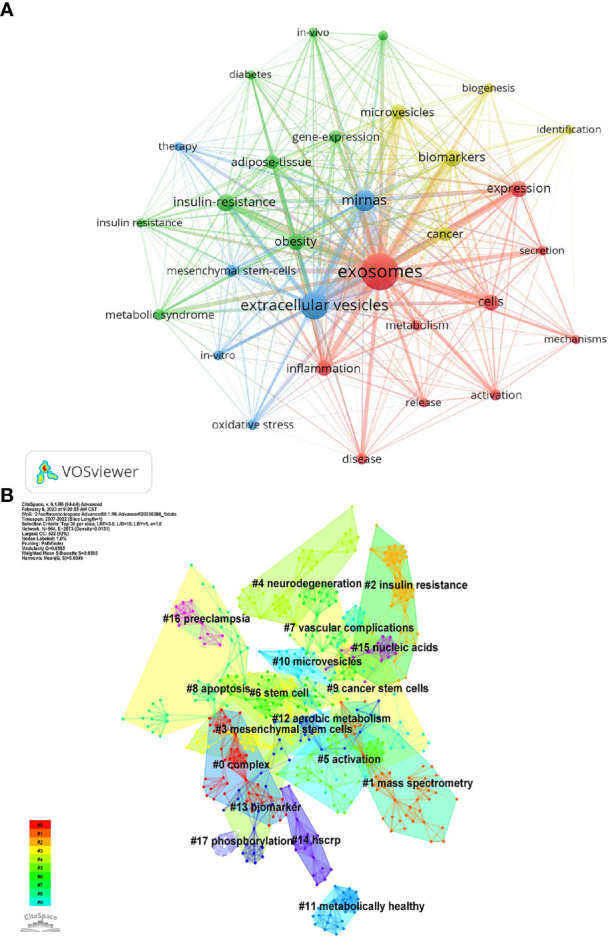
**(A)** Co-occurrence network of keywords by VOSviewer. **(B)** Keyword Cluster Analysis.

Keywords represent the central theme of a paper, and the keyword co-occurrence analysis can quickly capture the research hotspots in a certain field. **Table 5** shows the top 25 high-frequency keywords and the frequency of occurrence of exosomal research in metabolic diseases, among which microRNA (miRNA, miR) appeared 123 times, which is the hot spot for exosomes in metabolic diseases. The keywords were analyzed using VOSviewer (**Figure 6A**), and the threshold value was set at a minimum of 23 occurrences of the keywords, so a total of 29 keywords were identified, which were mainly divided into 4 clusters in different colors, representing different research directions. The keywords in the blue cluster include miRNAs, mesenchymal stem-cells, oxidative stress, etc.; the keywords in the yellow cluster include cancer, biomarkers, biogenesis, etc.; the keywords in the red cluster include metabolism, inflammation; the keywords in the green cluster include IR, adipose-tissue, obesity, etc.

**Table 5:** Top 25 keywords of documents on exosomes in metabolic diseases.

Rank	Keyword	Count	Rank	Keyword	Count
1	exosomes	346	14	mesenchymal stem-cells	40
2	extracellular vesicles	189	15	disease	34
3	mirnas	123	16	activation	33
4	biomarkers	79	16	metabolic syndrome	33
5	insulin-resistance	76	18	oxidative stress	32
6	expression	74	19	metabolism	31
7	obesity	70	20	in-vitro	29
8	cells	65	20	secretion	29
9	inflammation	61	22	in-vivo	28
9	microvesicles	61	23	biogenesis	27
11	cancer	57	23	circulating micrornas	27
12	adipose-tissue	50	25	identification	25
13	gene-expression	42	25	therapy	25

Using Citespace’s log-likelihood ratio-based algorithm to cluster the 18 keywords (**Figure 6B**) and further refining them, we can broadly group them into 5 categories, including related diseases (#2insulin resistance, #4neurodegeneration, # 7vascular complication, #11metabolically healthy, #12aerobic metabolism, #16preedampsia), related cells (#3mesenchymal stem cells, #6stem cell, #9cancer stem cells), cell biology-related (#5activation, #8apoptosis, #15nuclecic acids, #17phosphorylation), mass spectrometry-related (#0complex, #1mass spectrometry), and biomarkers (#13biomarker, #14hscrp).

**Figure 7:**
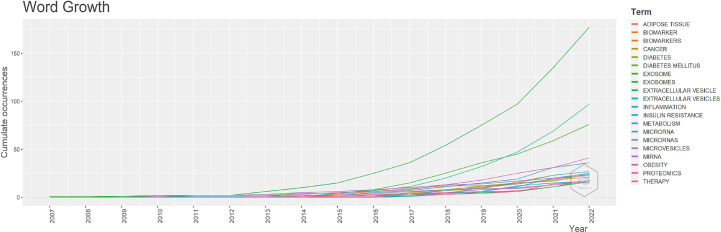
The cumulative growth keywords (top 20).

To study the hot trends, based on R-bibliometrix we conducted a word dynamics analysis. **Figure 7** shows the annual growth rate of the top 20 keywords, and we can see that all keywords started to increase from 2012. Among them, exosome shows a “j” curve, two keywords exosomes and miRNA also continue to grow quickly, the term diabetes mellitus been rapidly increasing since 2016, and the term IR grows at a slope about three times as high as before from 2020 onwards.

**Figure 8:**
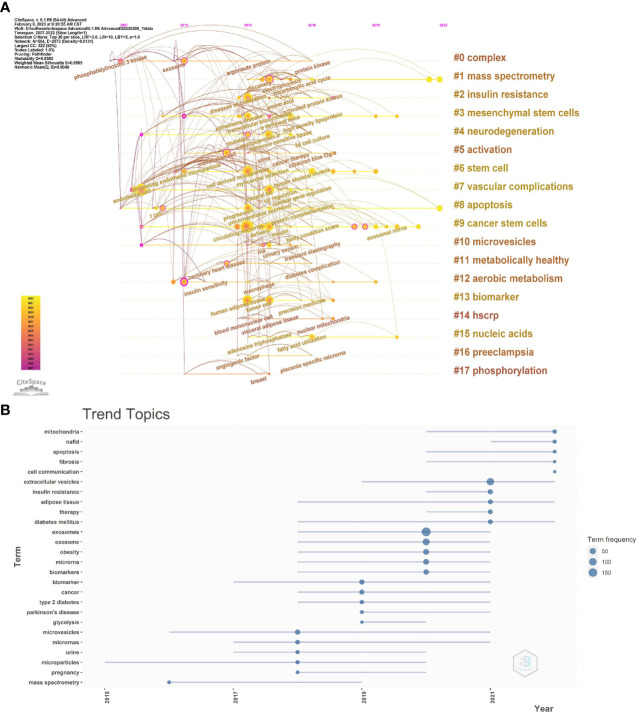
**(A)** CiteSpace visualization map of timeline viewer related to exosomes in metabolic diseases. **(B)** Trend topics. The X-axis represents the year, while the Y-axis is the cumulate occurrences of the keywords.

To further reflect the phase hotspots and developmental pathways of exosomes in metabolic diseases, we performed a timeline clustering of keywords using Citespace (**Figure 8A**) and a trend topic analysis using R-bibliometrix (**Figure 8B**). Combining the two figures, it can be seen that in 2007-2010, exosomes were less studied in metabolic diseases, focusing mainly on the cellular and extracellular vesicle levels; in 2010-2019, this theme received extensive attention, with studies focusing on specific molecular mechanisms associated with exosomes in different metabolic diseases, and the main keywords were insulin sensity, mutation, argonaute protein, protein kinase, transcellular biosynthesis, RNA, tricarboxylic acid cycle, etc.; after 2019, researchers began to explore exosomal exosome-associated miRNAs as well as the linkages between exosomes and mitochondrial, and they started to apply exosomes as biomarkers and therapeutics in metabolic diseases such as T2D, obesity, and NAFLD, which point the way to future research.

## 4 Discussion

### 4.1 General information

The objective of this article is to analyze the existing research literature on exosomes in metabolic diseases up to the year 2022. To achieve this goal, we utilized bibliometric techniques to examine the publication landscape with respect to trends, country and institutional distribution, journal distribution, and author distribution.

Research on exosomes in metabolic diseases has seen a gradual increase in annual publications since 2007, with a significant surge in interest over the past three years. China and the United States are the leading countries conducting research on this topic, together accounting for 60% of the global publications, with 80% of the top 10 institutions come from China and the United States. However, it is important to note that while the two countries have a strong collaborative relationship, China’s cooperation with other countries in this field is not as extensive as that of the United States. In addition, inter-institutional cooperation is mostly limited to domestic and lacks cross-country exchanges, which can hinder the development of this field.

The majority of literature on exosomes in metabolic diseases is currently published in journals related to molecular, cellular, immune, endocrine, and cancer research. Among these, the International Journal of Molecular Sciences is the most widely read journal in this field, while Theranostics has the highest impact factor. Co-cited journals are mostly Q1 journals with high impact and quality, providing a reliable theoretical foundation for future research and dissemination of research findings. However, it is important to note that current research on exosomes in metabolic diseases is primarily focused on basic research, and more efforts are needed to translate these research findings into clinical applications.

Among the top 10 authors and co-cited authors, Khalyfa Abdelnaby from the University of Missouri School of Medicine, USA has published extensively on the topic, with a specialization in biochemistry, molecular biology, and cell biology. His research focuses on the relationship between exosomes, sleep-disordered breathing, cardiovascular disease, and metabolic disorders (14–19). He emphasized the biological significance of exosomes and their role in various pathological conditions, highlighting the importance of their contents and transport. Notably, miRNAs circulating in exosomes can serve as functional biomarkers for diagnosis and outcome prediction, while synthetic miRNAs delivered through polymer-based nanoparticles are potential candidates for clinical therapy (19).

Thery C, from Paris Sciences & Lettres-PSL University in France, is the most highly cited co-cited author in the field of exosomes with 66 publications. With expertise in cell biology and immunology, Thery C has made significant contributions to the field of exosomes. In 1999, Thery C identified the selective accumulation of the heat shock protein hsc73 in dendritic cell-derived exosomes (20). In 2002, he published a review summarizing the composition, biogenesis, and function of exosomes (21), which he updated in 2014 (12, 22). In 2019, Thery C discussed the differences in properties between exosomes and other types of extracellular vesicles, highlighting their role as important mediators of intercellular communication (23). More recently, in 2021, he illustrated the properties of exosome versus small exosome secretion by tracking CD63 and CD9 intracellularly *in vivo* (24). These findings have laid a solid theoretical and experimental foundation for the study of exosomes in metabolic diseases.

### 4.2 Knowledge base

Co-cited literature is literature in the field that has been co-cited by multiple publications, and these articles generally deal with the characteristics, biological properties, classification, function of the research object and the important research mechanisms involved, which are seminal or summative for the field (25). The knowledge base is a collection of co-cited references that are frequently cited by researchers in a specific community, and it is not necessarily the same as highly cited references (26).In this study, we introduce the research base of exosomes in metabolic diseases by screening the top 10 co-cited references (**Table 4**).

The most frequently cited literature in the field of exosomes in metabolic diseases is a letter co-authored by Hadi Valadi and five other scholars, published in *Nature Cell Biology* in 2007 (10). The article reports on experiments using flow cytometry, microarray, and other techniques to demonstrate that exosomes contain mRNA and miRNA, which are transported to recipient cells to regulate their function, providing a novel mechanism for intercellular communication. Valadi et al. coined the term “exosomal shuttle RNA” to refer to these RNAs carried by exosomes.

Marina Colombo and two other researchers have published the second most co-cited literature in the *Annual Review of Cell and Developmental Biology* (12). In this review, the authors provide an illustrated definition of exosomes and other secreted extracellular vesicles, and discuss their isolation methods, biogenesis, secretion, and role in intercellular communication. Also, this paper links extracellular vesicles to infection biology, which has important implications for developing novel biomarkers, vaccines and therapeutics.

The third co-cited paper was published by Thomas Thomou et al. in *Nature* 2013 (11). In this paper the researchers demonstrated, using an adipose-specific knockout mouse model of the miRNA processing enzyme Dicer and the blood of humans suffering from lipodystrophy, that adipose tissue constitutes a major source of circulating exosomal miRNA that regulates mRNA expression, translation and systemic metabolism in distant tissues, resulting in a new class of adipokines.

The fourth co-cited review reveals the characteristics of exosomes and the mechanisms of their formation, targeting and function (13). The fifth co-cited paper shows through extensive experiments that adipose tissue macrophages secrete exosomes containing miR-155 carriers, which can be transferred to insulin target cells through paracrine or endocrine regulatory mechanisms to produce IR (27). The sixth co-cited paper, on the other hand, confirmed that exosome-like vesicles released from adipose tissue mediated the induction of tumor necrosis factor αand interleukin (IL)-6 and IR in macrophages *via* the TLR4/TRIF pathway (28).

The seventh co-cited review, published in 2018 by Guillaume van Neil et al. in *Nature Reviews Molecular Cell Biology*, with the highest impact factor (IF=113.915), describes the mechanisms involved in the intercellular communication of extracellular vesicles: cell classification, extracellular vesicle generation, and the interaction of extracellular vesicles with receptor cells (29). The eighth co-cited paper develops guidelines for minimal information on extracellular vesicle research in 2018 (30). The ninth reviews the physiological mechanisms of extracellular vesicles in bacteria, lower eukaryotes, and plants and discusses the molecular content and function of extracellular vesicles in a variety of tissues and body fluids, from cells to organs (31). The tenth article identifies proteins specifically enriched in small extracellular vesicles by proteomics and demonstrates the presence of exosomal and non-exosomal subpopulations in small extracellular vesicles (32).

The top 10 co-cited literature provide crucial insights into the characteristics, composition, biological functions, and target delivery of exosomes, thereby laying a strong foundation for the field.

### 4.3 Hotspots and frontiers

Keywords can help us quickly capture the distribution and evolution of hotspots in the field of exosomes in metabolic diseases. Based on keyword clustering and trend theme analysis, we summarize the current hotspots and future prospects of exosomes in metabolic diseases in the following areas.

#### 4.3.1 Exosomes-producing parent cells

Adipocyte-derived exosomes are the most studied among metabolic diseases. Adipocytes in adipose tissue secrete exosomes that play an important role in influencing systemic glucolipid metabolism. *In vivo*, visceral adipocyte-derived exosomes in obese individuals carry miRNAs that target the transforming growth factor(TGF)-β and classical Wnt signaling pathways, and play a role in fat distribution, adipocyte differentiation, end-organ inflammation and fibrosis signaling (33); exosomes secreted by visceral adipose tissue inhibit ATP-binding cassette transporter A1(ABCA1) by transporting miRNAs, allowing cholesterol to accumulate in macrophages and promotes foam cell production, leading to lipid plaque formation (34). *In vitro* experiments, injection of adipocyte-derived exosomes from obese mice induced IR in normal mice (28); exosomes isolated from visceral adipose tissue of obese mice induced by high-fat diet promoted macrophage foaminess and M1 macrophage polarization by down-regulating ABCA1 and ATP-binding cassette transporter G1-mediated cholesterol efflux and up-regulating nuclear factor κB(NF-κB) activity (35). Clinical studies have also shown that exocrine secretion from circulating adipocytes reduces IR to maintain glucose homeostasis, which may account for gastric bypass surgery and subsequent weight loss (36).

Macrophage-derived exosomes have been a hot topic in metabolic diseases in recent years. Macrophages belong to the monocyte lineage and are closely associated with the inflammatory response of tissues. Macrophages secrete exosomes that inherit the functions of the parent cell and regulate immune and inflammatory responses by delivering immunoregulatory-related proteins, nucleic acids and lipids from macrophages to downstream cells (37). *In vitro*, studies have found that lipoprotein-treated macrophage exosomes activate naïve macrophage NF-κB signaling pathways and induce the secretion of inflammatory factors and chemokines, thereby promoting inflammatory responses (38). When macrophages were stimulated with high concentrations of glucose, TGF-β1 mRNA was highly expressed in exosomes from stimulated macrophages, and the exosomes transmitted TGF-β1 mRNA to glomerular thylakoid cells, inducing them to secrete more tumor necrosis factor α, IL-1β and monocyte chemotactic protein 1 to promote the inflammatory response and stimulate glomerular thylakoid cell proliferation (39); meanwhile, high glucose-stimulated macrophage exosomes contain high concentrations of IL-1β and inducible nitric oxide synthase, which stimulate NF-κB signaling pathway to induce naive macrophage differentiation to release pro-inflammatory cytokines (40). *In vivo*, macrophage foam cells release large amounts of exosomes that activate the ERK and AKT pathways in vascular smooth muscle cells (VSMCs), promoting migration and adhesion of VSMCs (41); human monocyte macrophage exosomes also activate the NF-κB pathway leading to endothelial cell dysfunction and AS (42). Recently, Adipose tissue macrophage-derived exosomes have also been shown to be important mediators in the regulation of adipose tissue function and insulin sensitivity. It has been reported that when exosomes secreted by adipose macrophages from obese mice were injected into lean mice caused poor glucose tolerance and IR; in contrast, when exosomes obtained from lean mice were injected into obese mice improved glucose tolerance and insulin sensitivity (27).

In addition, exosomes of different cellular origin are of interest in AS. Exosomes of endothelial origin can modulate vascular pathology by promoting vascular injury, inducing endothelial dysfunction and vascular inflammatory responses, interfering with coagulation pathways, and regulating vascular homeostasis (43). Exosomes generated by VSMCs produce proatherogenic effects through endothelial dysfunction and vascular calcification (44). And exosomes produced locally by platelets in small arteries or arterioles can promote coagulation cascades by providing and expanding the response to acute arterial obstruction (45).

Also noteworthy are the exosomes produced by the gut microbiota (46), which are involved not only in IR and impaired glucose metabolism promoted by a high-fat diet (47), but also in altering clock expression in target tissues to disrupt circadian rhythms by inducing systemic inflammation and metabolic disturbances (14). However, the extent to which gut microbiota contributes to the pathogenesis of metabolic diseases through the action of exosomes remains uncertain.

Taken together, different parent cells-derived exosomes are involved in the processes of metabolic diseases through multiple mechanisms.

#### 4.3.2 Cargos in exosomes

Exosomes contain a variety of bioactive substances such as lipids, proteins and nucleic acids. miRNAs are currently the most studied substances in exosomes(**Table 6**).

**Table 6:** Important functional microRNAs in exosomes in metabolic diseases.

miRNA	diseases	source	function
miR-155 (24, 45)	T2D	macrophages in adipose tissue	causes poor glucose tolerance and insulin resistance
miR;-29a (46)		macrophages in adipose tissue	causes poor glucose tolerance, insulin resistance and affects insulin release
miR-27a (47, 48).		adipose tissue	regulates insulin sensitivity in skeletal muscle
miR-210 (49)		macrophages	inhibits glucose uptake and mitochondrial function
miR-375 (50, 51)		serum and plasma	reduce insulin secretion
miR-155 (52).	AS	VSMCs	increases permeability of ECs and promotes AS plaque formation
miR-155 (53)		ECs	enhance monocyte activation
miR-146a (54)		macrophages	reduces macrophage migration and promotes macrophage adhesion in the vessel wall
miR-143, miR-145 (55)		ECs	regulates the conversion of synthetic VSMCs to contractile ones in AS plaques
miR-25-3p (56)		platelets	inhibits inflammation and lipid deposition in ECs
miR-223 (57)		platelets	inhibits endothelial inflammation
miR-122 (58)	NAFLD	hepatocytes	involves in free cholesterol transport and high-density lipoprotein anabolism
miR-143 (59)		hepatocytes	induces the inactivation of the insulin-induced AKT pathway in the liver
miR-23b, miR-148b, miR-4269, miR-4429 (60)		visceral adipocytes	leads to the progression of NAFLD to hepatic fibrosis
miR-128 (61)		hepatocytes	

T2D is closely associated with miR-155, miR-29a, miR-27a, miR-210 and miR-375. In obese mice, miR-155 and miR-29a were highly expressed in the exosomes secreted by macrophages in adipose tissue, they caused poor glucose tolerance and IR in healthy mice by interfering with cellular insulin signaling (27, 48). Moreover, miR-29a is also transmitted by macrophage exosomes to pancreatic β-cells to affect insulin release (49). Adipose tissue secreted exosomes carry miR-27a into circulating blood and remotely regulate insulin sensitivity in skeletal muscle; miR-27a also induces IR in skeletal muscle cells by regulating peroxisome proliferator-activated receptor γ and its downstream genes (50, 51). In addition, high concentrations of glucose stimulate macrophages to secrete exosomes, which transmit cellular signals by carrying miR-210 and inhibit glucose uptake and mitochondrial function in adipocytes (52). Exosomes in serum or plasma enriched with miR-375 regulate actin function by inhibiting MTPN to reduce insulin secretion (53, 54).

AS is associated with multiple miRNAs that have both AS-promoting and AS-inhibiting effects, respectively. miR-155 levels are significantly elevated in exosomes secreted by VSMCs, which enter endothelial cells (ECs) and disrupt ECs tight junctions and endothelial barrier integrity by regulating the targeting of tight junction proteins ZO-1 and claudin 1. This leads to increased permeability of ECs and promotes AS plaque formation (55). Exosomes secreted by ECs also carry miR-155, and these exosomes are transferred to human monocyte THP1 cells, shifting the monocyte/macrophage balance from anti-inflammatory M2 macrophages to pro-inflammatory M1 macrophages to enhance monocyte activation (56). Macrophage exosomes are enriched in miR-146a and accelerate the development of AS by reducing macrophage migration and promoting macrophage adhesion in the vessel wall through the target proteins IGF2BP1 and HUR (57). On the other hand, exosomes also release miRNAs with protective effects. Under high shear stress, ECs release miR-143- and miR-145-rich exosomes that downregulate KLF4/5, ELK1, and CAMK2D *via* miR-143/145, thereby regulating the conversion of synthetic VSMCs to contractile ones in AS plaques. VSMCs can also release miR-143/145-rich exosomes on their own, which play a key role in the differentiation of VSMCs (58). miR-25-3p in platelet exosomes downregulates IL-6, IL-1β and TNF-ɑ levels and inhibits oxidized low-density lipoprotein induced inflammation and lipid deposition in ECs; it also inhibits NF-κB signaling pathway and inflammation in ECs by targeting Adam10 (59). In addition, thrombin-activated platelets can carry miR-223 into ECs *via* exosomes and downregulate ICAM-1 expression, and may inhibit endothelial inflammation by regulating NF-κB and MAPK pathways as well (60).

Exosome-carrying miRNAs have been less studied in NAFLD. Currently, it has been found that hepatocytes synthesize miR-122, which is transported *via* exosomes and binds to sterol regulatory element binding protein 2, and is involved in free cholesterol transport and high-density lipoprotein anabolism (61). Exosomes secreted by hepatocytes were also enriched in miR-143, which reduced the expression of oxygen sterol-binding protein-related protein 8, thereby inducing the inactivation of the insulin-induced AKT pathway in the liver (62). Visceral adipocyte-derived exosomes from obese individuals carry miR-23b, miR-148b, miR-4269, and miR-4429 into hepatocytes and upregulate the expression of TIMP-1, TIMP-4, Smad-3, and MMP-9, thereby inducing abnormal regulation of the TGF-β signaling pathway and leading to the progression of NAFLD to hepatic fibrosis (63). It was also found that hepatic stellate cells can effectively internalize miR-128-3p in hepatocyte exosomes, thereby promoting the development of hepatic fibrosis by inhibiting peroxisome proliferators-activated receptor (PPAR)-γ expression (64).

The role of proteins in exosomes in metabolic diseases has not been extensively studied. Chen et al. reported that macrophage-derived exosomes carrying high mobility group protein 1, a nuclear non-histone DNA binding protein, directly impair insulin signaling in adipocytes cultured *in vitro* (65). Ibrahim et al. found that lysophosphatidylcholine induced hepatocytes to secrete exosomes containing c-x-c motif chemokine 10, which is chemo-attractive to macrophages *in vitro* (66). Kakazu et al. reported that stimulation of hepatocytes with palmitic acid releases C16:0 ceramide-rich exosomes that are chemotactic to macrophages (67). Additionally, Poverod et al. discovered that lipotoxicity-induced hepatocytes release exosomes rich in vascular non-inflammatory molecule-1, a surface cargo protein, which induces pro-angiogenesis in endothelial cells (68).

In summary, exosomes carrying cargos are released from parental cells and target recipient cells, enabling intercellular crosstalk in metabolic diseases.

#### 4.3.3 Target cells and effects

Exosomes, protected by their lipid bilayer, are stable in circulation and can target specific sites to mediate biological processes and participate in the effector mechanisms of disease.

Adipose tissue, liver, and muscle are insulin target organs that play a crucial role in promoting glucose uptake and inhibiting lipid hydrolysis. When IR occurs in the body, insulin signaling (AKT-GSK signaling pathway, etc.) will be inhibited, which in turn will hinder a series of biological processes such as downstream glycogen synthesis, protein synthesis, glucose transport and anti-lipolysis, leading to the development of metabolic diseases such as obesity, T2D and NAFLD. Exosomes and the cargos they carry are secreted from parental cells and act on target cells such as adipocytes, hepatocytes and skeletal muscle cells to participate in the IR process. In obese mice, adipocytes, hepatocytes and muscle cells are regulated by exosomes secreted by adipose tissue macrophages. miR-155 within exosomes downregulates the phosphorylated AKT signaling pathway and inhibits the expression of intracellular lipogenic transcription factors PPAR-γ and CCAAT/enhancer binding protein β, thereby exacerbating IR (69). Adipocytes are also stimulated by exosomes released from hypoxic adipocytes, which reduce the uptake of 2-deoxyglucose and impair insulin sensitivity (70). Hepatocytes are also stimulated by exosomes derived from human subcutaneous or visceral adipocytes, which inhibit AKT phosphorylation and downregulate the expression of insulin receptor substrate 1 and hormone-sensitive adiponectin in adipocytes, promoting IR (50, 71). In addition, as the producer of insulin, pancreatic β-cells are important target cells as well. β-cells are regulated by miR-29a in macrophage exosomes and miR-375 in their own exosomes, which in turn affects cellular insulin release (49, 72). In a mouse model of IR induced by a high-fat diet, β cells proliferate following regulation by skeletal muscle cell-derived exosomes, which explains the adaptation of β-cell mass during IR (73).

Metabolic diseases are often accompanied by chronic inflammation, which is a significant contributing factor to their pathogenesis, such as AS, which is a chronic inflammatory disease caused by intravascular lipid accumulation. In the inflammatory response, monocyte-macrophages play an essential role in both pro-inflammatory and anti-inflammatory processes. In AS, monocyte-macrophages, stimulated by adipocyte-derived exosomes, polarize toward the M1 type and release the inflammatory cytokines TNF-α, IL-6, TLR4/TRIF, etc. (72). Macrophages, stimulated by hepatocyte-secreted exosomes, are recruited in large numbers and activated as M1 type macrophages, producing large amounts of pro-inflammatory cytokines to promote nonalcoholic steatohepatitis (74). It has also been shown that the activation of M1 macrophages in obese mice is mediated by the exosome-associated protein, sonic hedgehog activates the Ptch/PI3K signaling pathway (28). In contrast, monocyte-macrophages are stimulated by exosomes secreted by Adipose-derived stem cells to polarize toward the M2 type, triggering white adipose tissue browning and reducing inflammation (75). Neutrophils also play an important role in the pro-inflammatory response. Neutrophils are induced to secrete large amounts of inflammatory cytokines such as IL-1β, TNF-α, and IL-6 by exosomes secreted by oxidized low density lipoprotein-stimulated macrophages, while upregulating intracellular reactive oxygen species production, exacerbating the inflammatory response in AS (72).

The NAFLD disease process includes simple steatohepatitis, non-alcoholic steatohepatitis, liver fibrosis and cirrhosis, and the progression of NAFLD is characterized by liver inflammation and fibrosis following repeated and persistent injury. We have already mentioned the inflammatory response above, below we will focus on the main target cells of liver fibrosis - hepatocytes and hepatic stellate cells. Stimulation of hepatocytes by adipose tissue-derived exosomes leads to dysregulation of the TGF-β pathway (63), whereas stimulation of hepatic stellate cells by adipose tissue-derived exosomes upregulates the hepatic extracellular matrix (ECM) expression of fibrinogen activator inhibitor-1, matrix metalloproteinase (MMP)-7 and tissue inhibitor of metalloproteinases-1 and inhibits PPAR -γ expression to promote liver fibrosis (76, 77).

To sum up, the secretion of cargo-carrying exosomes from parental cells acting on target cells exerts effects in metabolic diseases, making an important addition to the pathogenesis of metabolic diseases.

#### 4.3.4 Biomarkers

In recent years, the development of isolation and purification techniques has enabled the isolation of exosomes from almost all body fluids, including serum, plasma, urine, milk, and saliva, opening up a new era in disease diagnosis. Many miRNAs have been identified as potential biomarkers for the diagnosis and prognosis of metabolic diseases. In contrast, it was found that exosome-encapsulated miRNAs may be biologically more active and relevant than vesicle-free miRNAs.

In an experiment, Lakhter et al. found that β-cells released miR-21-5p-rich exosomes in response to cytokine stimulation and detected a threefold increase in miR-21-5p levels in exosomes in the sera of children with new-onset T1DM compared to healthy children (78). It was also found that exosomes from urine of patients with diabetic nephropathy (DN) were detected to contain high amounts of miR-130a and miR-145, and these miRNAs have been shown to be exosomes derived from glomerular thylakoid cells. Furthermore, in cellular experiments, there was a positive correlation between glucose concentration and the number of exosomes as well as miR-145 expression (79). Another investigator found that miR-362-3p, miR-877-3p and miR-150-5p were upregulated and miR-15a-5p was downregulated in urinary exosomes from diabetic patients. These miRNAs may regulate DN through p53, mTOR and AMPK pathways and are potential noninvasive markers of early DN (80). Rossi et al. reported that water channel protein (AQP) 5 and AQP2 were detected significantly increased in urinary exosomes of DN patients as well as 35 diabetic patients, they were expressed on epithelial renal tubular cell membranes and correlated with the histological grading of DN (81). A meta-analysis also reported significantly higher levels of exosomes of platelet, endothelial and monocyte origin in T2D patients from 48 independent studies, and their numbers were positively correlated with BMI, Homeostasis model assessment (HMA)-IR and HOMA-β, which was used to evaluate beta-cell function (82, 83). Furthermore, in studies of gestational diabetes mellitus, plasma concentrations of exosomes were found to increase progressively with increasing duration of gestation and more markedly in gestational diabetes mellitus (2.2-fold, 1.5-fold and 1.8-fold greater than normal gestation at three-time points, respectively), which may make exosomes an effective diagnostic tool for screening susceptible individuals in the future (84). In summary, exosomes isolated from body fluids and their cargos are expected to be effective biomarkers for diabetes and its complications.

Wang et al. found elevated levels of miR-30e and miR-92a in plasma exosomes of AS patients compared to healthy individuals, which regulate cholesterol metabolism by targeting ABCA1 and thus are expected to be new biomarkers of AS (85). Povero et al. isolated exosomes from a high-fat diet NAFLD mouse model and found that exosomes carried different proteins than controls and had increased levels of miR-122 and miR-192 in the blood and decreased proteins in the liver. In addition, bruno et al. found a positive correlation between miR-192 levels in exosomes of patients’ blood and NAFLD liver inflammatory activity scores and disease progression in a clinical trial (74). These experimental results suggest that these miRNAs may be a prospective marker reflecting the development of fatty liver disease (86). Moreover, miR-122 also plays an important role in the study of liver fibrosis, and reducing its expression may induce downregulation of some liver reconstitution regulators such as mitogen-activated protein kinase 3, which could reflect the extent of liver regeneration under pathological conditions (87). In a study by Chen et al., miR-122a was found to fluctuate with BAT activity in mice, suggesting that it, along with miR-92a associated with exosomes, may serve as a serological marker reflecting BAT activity in humans (88).

#### 4.3.5 Treatment

Exosomes have a natural lipid bilayer, low immunogenicity and circulating stability, and also contain surface proteins recognized by target gene cells, so that exosomes can be used as vehicles for drug delivery and thus can fuse with the plasma membrane and transport specific substances to act on target cells (89).

Adipose stem cell-derived exosomes were found to carry active signaling and STAT3 proteins that induce the conversion of obese mouse macrophages to anti-inflammatory M2 phenotype, thus alleviating IR and metabolic disorders in obese mice (75). Additionally, Tsukita et al. reported that exosomes secreted from mouse bone marrow cells containing miR-106b-5p and miR-222-3p promoted pancreatic β-cell proliferation by downregulating the cell cycle blocking proteins pathway, ultimately improving hyperglycemia in diabetic mice following bone marrow transplantation (90).

Mesenchymal stem cells(MSCs) are pluripotent cells that possess the potential to repair damaged tissues, modulate immune responses, and induce angiogenesis; MSCs regulate the biological functions of various tissue cells by secreting exosomes that transduce a variety of signaling molecules and have therapeutic effects on a variety of metabolic diseases, including AS (91). Exosomes derived from MSCs contain high levels of a variety of miRNAs that inhibit AS plaque formation and a variety of proteins that inhibit inflammation and promote extracellular matrix synthesis (92). Specifically, MSCs-derived exosomes promote macrophage M2 polarization in AS plaques and inhibit macrophage infiltration, thereby reducing plaque volume (93). Adipose-derived MSCs secreted exosomes to inhibit miR-342-5p through protein phosphatase 1 regulatory subunit 12B, which effectively protected endothelial cells from AS (94). miRNA-221 carried by MSC-secreted exosomes reduced lipid deposition in mouse aortic tissue by downregulating NAT1 and inhibited IGF2/IGF2R signaling pathway activation (95), thereby inhibiting the differentiation of MSCs and SMCs toward osteogenesis and apoptosis of vascular smooth muscle cells, ultimately resulting in the inhibition of AS plaque formation (96). Moreover, exosomes secreted from rat bone marrow (BM)-MSCs can repair damaged neurons and astrocytes and reverse dysfunction, making them promising therapeutic tools for treating diabetic nerve injury, as found by Nakano et al. (97).

**Figure 9:**
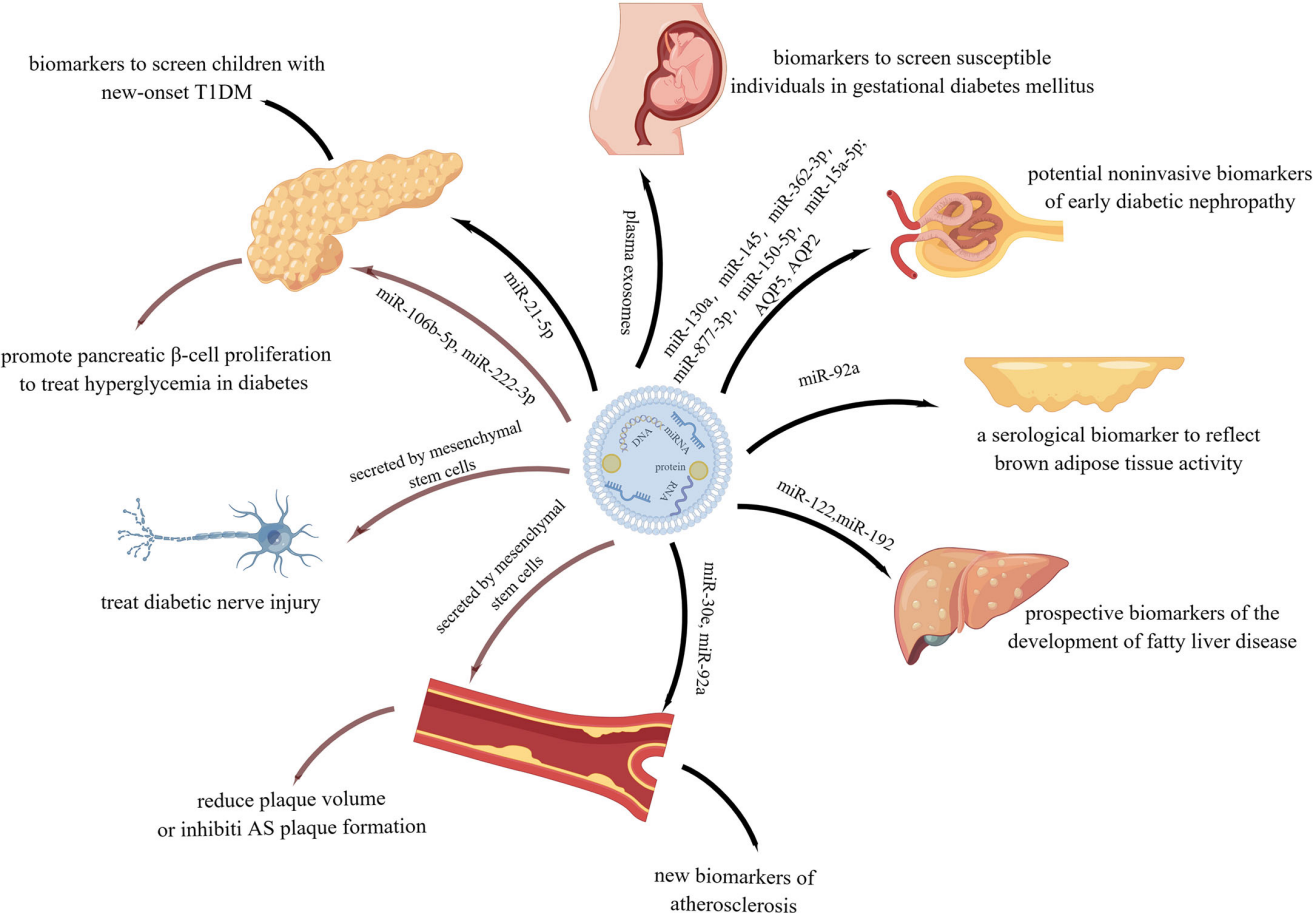
Function of exosomes in metabolic diseases in diagnosis and treatment.

In conclusion, exosomes not only play a role in the occurrence and development of many metabolic diseases, but also serve as biomarkers in their diagnosis and as therapeutic carriers in their treatment (**Figure 9**). Therefore, exploring the production, properties, roles, and mechanisms of exosomes holds great potential in the field of metabolic diseases.

## 5 Limitations

This study extracted the relevant literature on exosomes in metabolic diseases from the WoSCC database and used three bibliometric tools simultaneously for the analysis, but there are still some limitations to this study. Firstly, the data were obtained from a single database because we are unable to undertake relevant analysis (such as co-citation analysis) on PubMed or other databases (lack of information on references) due to software limitations. As a result, this study may have some bias and less comprehensive and accurate findings. Secondly, VOSviewer, CiteSpace, and bibliometrix cannot completely replace systematic searching, so a more accurate literature analysis should be based on software analysis and combined with specific literature to construct a knowledge graph. Nonetheless, visualization-based literature analysis can still provide effective help for researchers to understand hotspots and potential problems in exosomes in metabolic diseases.

## 6 Conclusion

Exosomes are of increasing significance in metabolic diseases in terms of both research and application potential. This area of research is developing steadily, with active international collaboration, particularly between China and the United States. Khalyfa, Abdelnaby has made significant contributions to many relevant publications and Thery C has the most co-citations in the study. The current research is mainly focused on basic research on cells and miRNAs, so attention should also be given to translating research findings into clinical applications for the diagnosis and treatment of metabolic diseases using exosomes. In conclusion, this study provides the first systematically bibliometric analysis of publications related to exosomes in metabolic diseases, offering an objective and comprehensive overview of the field and a valuable reference for researchers in the field.

## Data availability statement

The original contributions presented in the study are included in the article/**Supplementary material**. Further inquiries can be directed to the corresponding authors.

## Author contributions

FX and LD designed this study, FX collected the data and performed the analysis, and CX normalized the pictures. FX and CX wrote the original draft, and XH revised the manuscript. All authors contributed to the article and approved the submitted version.
