# Supplementary material for: Knowledge mapping of exosomes in metabolic diseases: a bibliometric analysis (2007-2022)
Source: Front Endocrinol (Lausanne). 2023 May 8;14:1176430. doi: 10.3389/fendo.2023.1176430 (PMC10200891; doi:10.3389/fendo.2023.1176430)
Supplement: Supplementary file 1 [file Image_1.pdf]

Supplementary Material

Knowledge Mapping of Exosomes in Metabolic Diseases: A Bibliometric Analysis (2007-2022)

Fangzhi Xu1 †, Chenxi Xia2 †, Lin Dou1*, Xiuqing Huang1*

*** Correspondence:** Lin Dou1* doulin4623@bjhmoh.cn

Xiuqing Huang1* huangxiuqing3768@bjhmoh.cn

1.1 Supplementary Figures

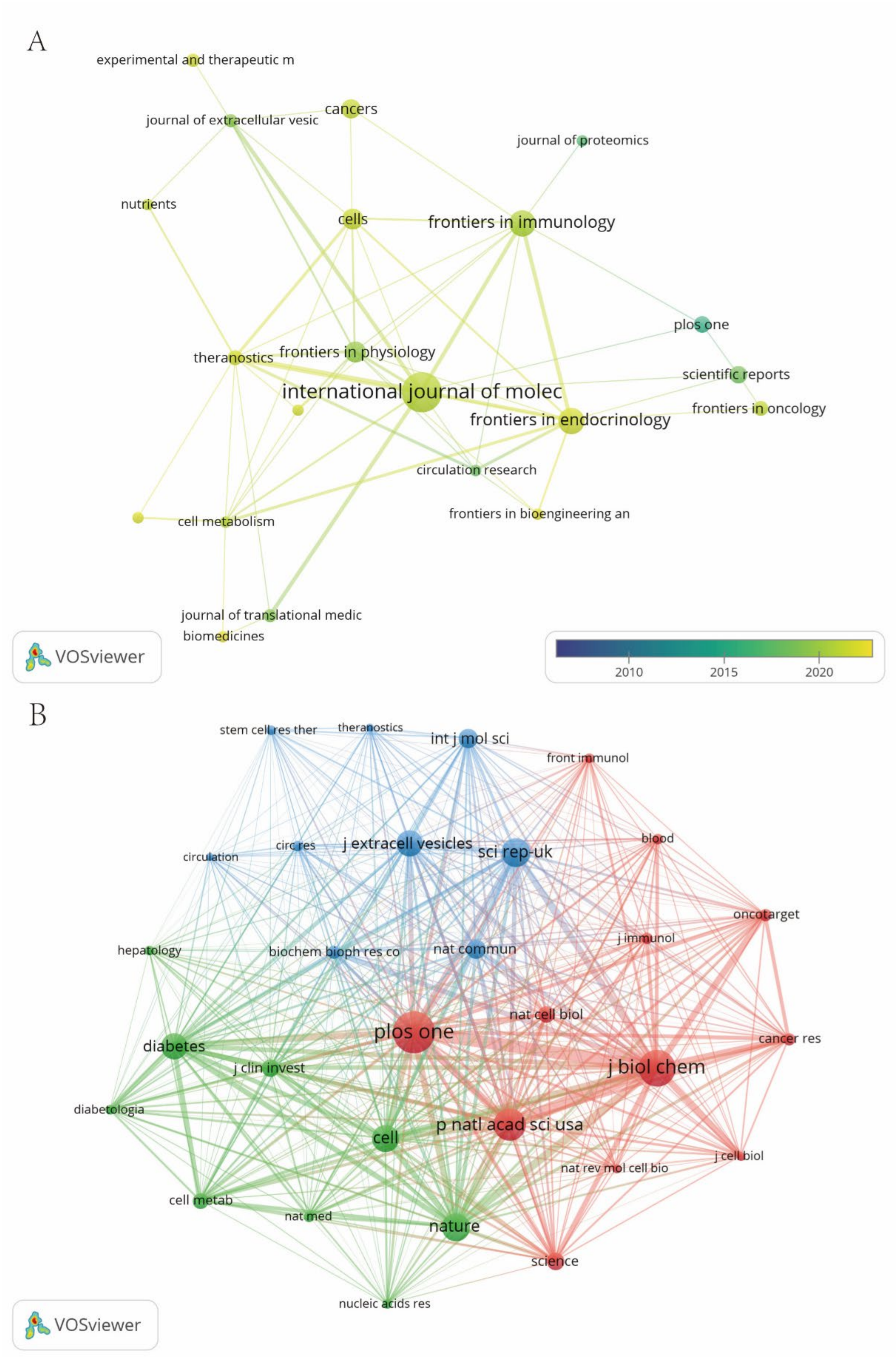

Supplementary figure 1
The distribution of journals publishing research on exosomes in metabolic diseases. (A) The visualization of journals, (B) The visualization of co-cited journals.

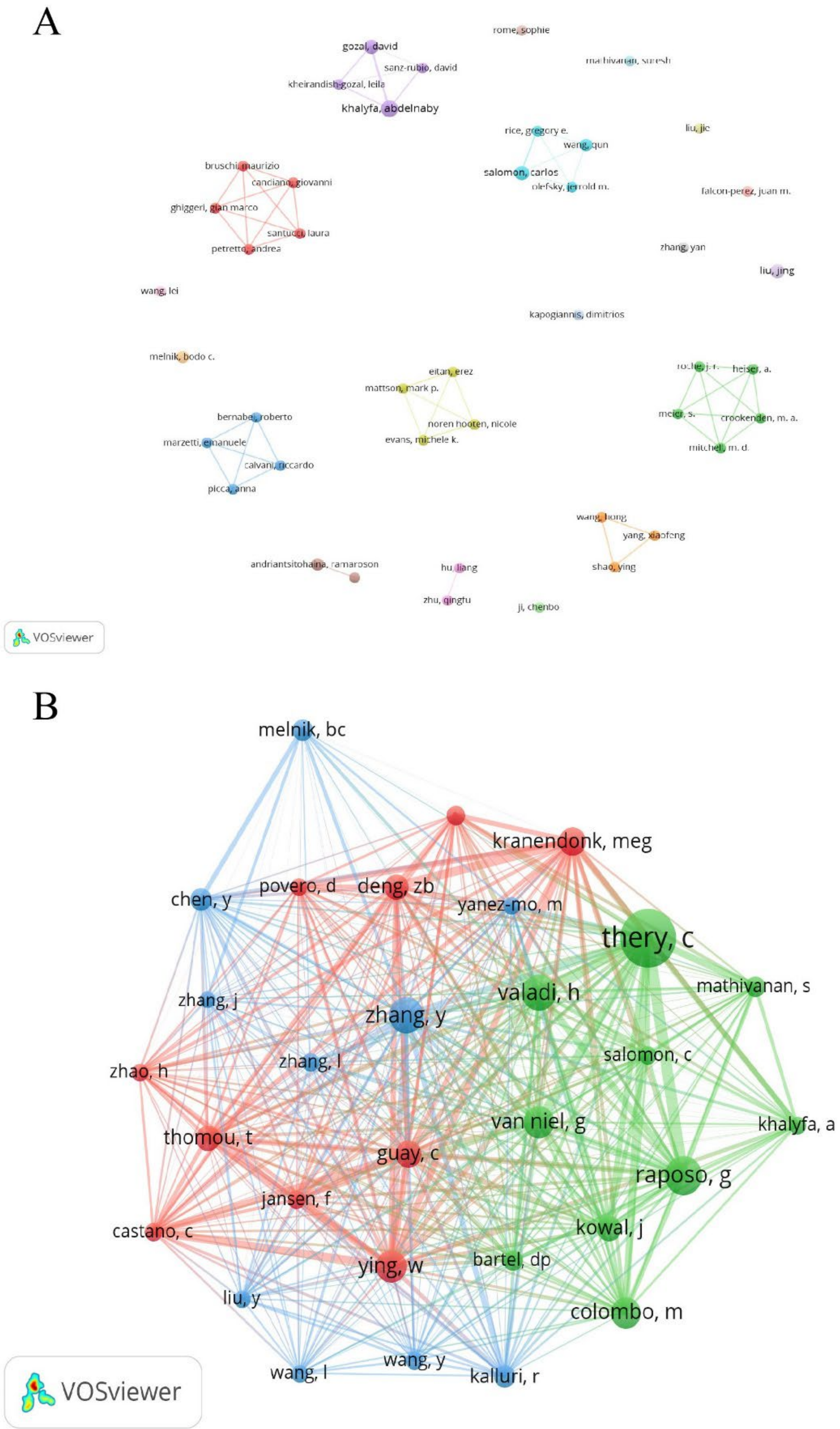

Supplementary figure 2

The visualization of authors (A) and co-cited Authors (B) on research of exosomes in metabolic diseases.
